# Burden of Inadequate Physical Activity Among Adults in a Rural Area of Central India

**DOI:** 10.7759/cureus.114661

**Published:** 2026-08-17

**Authors:** Piyushi V Patil, Meenal V Kulkarni

**Affiliations:** 1 Community Medicine, N.K.P. Salve Institute of Medical Sciences and Research Centre and Lata Mangeshkar Hospital, Nagpur, IND

**Keywords:** cross-sectional study, ipaq, non-communicable diseases, physical inactivity, rural population

## Abstract

Background

Physical inactivity is a principal modifiable behavioral risk factor responsible for the continuing rise of non-communicable diseases. Many adults do not meet the recommended levels of physical activity, and the prevalence varies between populations. The present study aimed to ascertain the prevalence of inadequate physical activity among adults in a rural area of central India and its association with selected sociodemographic factors and body mass index.

Method

A community-based analytical cross-sectional study was conducted among 400 adults selected by simple random sampling. Physical activity was assessed using the short form of the International Physical Activity Questionnaire. Descriptive statistics and chi-square test were used for statistical analysis.

Results

The prevalence of inactivity was found to be 54.75% (n=219). Females were more likely to have low physical activity levels than males (71.9% vs. 37.8%; p<0.001). No associations were found between physical activity and the other sociodemographic variables.

Conclusion

Half or more of the rural adult population has an inadequate level of physical activity, as exhibited in a much higher frequency than in males, especially among females. The results suggest the importance of community-based interventions to increase physical activity, with an emphasis on women as an at-risk group.

## Introduction

Non-communicable diseases (NCDs) are an increasing public health burden globally and are responsible for a vast majority of premature deaths and disability-adjusted life years, notably in low- and middle-income countries [1,2]. Physical inactivity is an important modifiable behavioral risk factor of cardiovascular diseases, type 2 diabetes mellitus, certain malignancies, obesity, depression, and shorter life expectancy [2]. To help mitigate the risk of NCDs, the World Health Organization (WHO) recommends that adults should perform at least 150-300 minutes a week of moderate-intensity aerobic physical activity, or 75 to 150 minutes a week of vigorous-intensity activity [3]. Regular physical activity provides health benefits with respect to metabolic control, cardiovascular fitness, and psychological well-being. Yet a significant global number of persons are presently insufficiently active [4]. More than one-quarter of adults worldwide do not reach the recommended levels, and much higher levels have been observed in regions with rapid transition [3,5]. The mechanization of work, increasing use of motorized transport, dependence on technology, and sedentary leisure pursuits have led to less active lifestyles in many regions [6].

In India too, physical inactivity is being recognized as an important public health challenge. In the Indian Council of Medical Research-India Diabetes (ICMR-INDIAB) study covering 11 states, small proportions of adults engaged in leisure-time physical activities [7]. More recently, pooled data from the Lancet Global Health study showed that 49.4% of adults in India were insufficiently physically active in 2022, a large increase over earlier estimates. Women appear to be significantly less active than men, and the study shows that even the rural population, who are not traditionally considered sedentary, are now becoming so [8]. Since culture, occupation, and huge individual variability make population measurement of physical activity difficult, WHO also launched a project to develop a standard index widely used in population surveillance of physical activity. The short form of the International Physical Activity Questionnaire (IPAQ-SF) is a standardized instrument that has demonstrated acceptable reliability and validity across diverse populations [9]. Community-level data are sparse in rural regions of India, which could help in developing locally appropriate interventions.

The present study aimed to ascertain the prevalence of inadequate physical activity among adults in a rural area of central India and its association with selected sociodemographic factors and body mass index (BMI).

## Materials and methods

A community-based analytical cross-sectional study was conducted in a rural field practice area of the Department of Community Medicine (attached to a medical college in central India). The study population comprised adults residing in the selected village. Ethical clearance was obtained from the Institutional Ethics Committee (Approval No. NKPSIMS & RC and LMH/IEC/5/2024) before commencement of the study. Verbal informed consent was obtained from all participants before data collection, as approved by the Institutional Ethics Committee. All adults aged 18 years and above residing in the selected village were eligible for inclusion. Individuals who were bedridden, severely ill, or not available at the time of the survey were excluded from the study (Table 1). The sample size was calculated based on the prevalence of physical inactivity from the most recent pooled analysis published in the Lancet Global Health [8]. Using a single-proportion formula, at a 95% confidence interval (CI) and an absolute precision of 5%, the minimum sample size was estimated at 381. The sample size was then rounded up to 400 participants for feasibility and study power. A total of 10 villages from the rural field practice area were considered for this study, out of which one village was selected using simple random sampling. A list of the households in that village was procured from the local records. The selection of households was conducted using systematic random sampling, and one eligible adult from the selected households was chosen at random for the study.

**Table 1:** Inclusion and exclusion criteria

Inclusion criteria	Exclusion criteria
Adults aged 18 years and above	Bedridden individuals
Permanent residents of the selected village	Severely ill individuals
	Not available at the time of the survey

Data collection

A house-to-house survey was carried out for data collection. Data were collected using a predesigned and pretested questionnaire, including sociodemographic variables. Physical activity was assessed using the IPAQ-SF, a standardized and validated instrument for assessing physical activity in population-based studies [9]. The IPAQ-SF is a publicly available, open-access instrument distributed under a Creative Commons Attribution (CC BY 4.0) license; it may be used for research without obtaining separate permission, provided that it is appropriately attributed. This condition was met by citing the source instrument; accordingly, no additional permission or license fee was required for its use in the present study. Physical activity levels were categorized as low, moderate, or high according to the official IPAQ scoring protocol. As per the scoring protocol, respondents were classified as low, moderate, or high as per the duration and intensity of activity performed in a week. BMI was calculated and classified according to the WHO classification. 

Statistical analysis

Data were entered and analyzed using Stata Statistical Software, Release 10.1 (StataCorp LP, College Station, TX, USA). Descriptive statistics were used to summarize participant characteristics. Associations between physical activity level and categorical variables were assessed using the chi-square test. p-value <0.05 was considered statistically significant.

## Results

**Figure 1:**
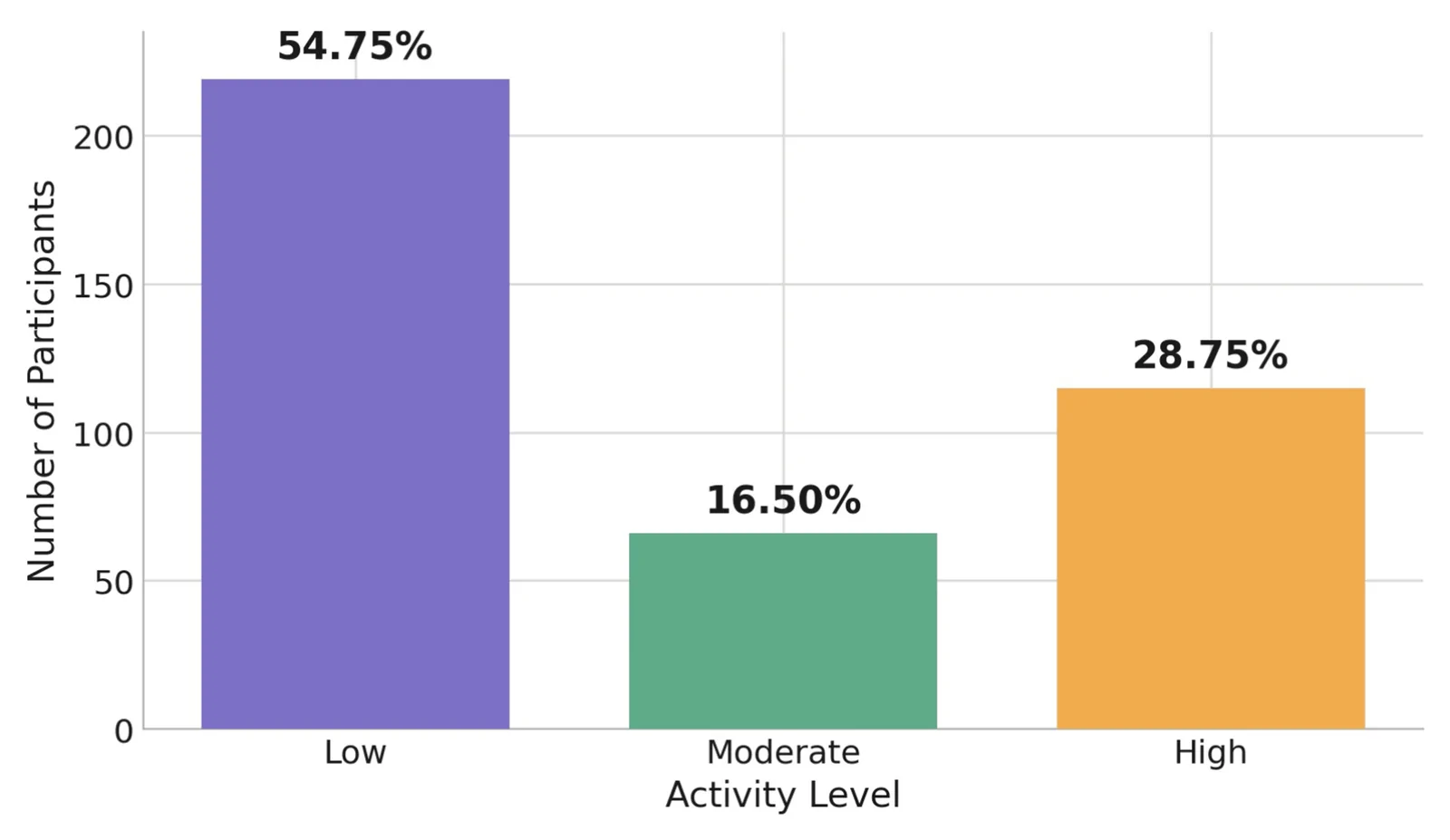
Level of physical activity of the participants (n=400)

A total of 400 participants were included in the study, comprising 201 (50.25%) males and 199 (49.75%) females. The largest proportion belonged to the 30-44 years age group (n=135, 33.75%). In terms of educational status, 114 participants (28.5%) had completed lower secondary education, while 141 (35.25%) were classified under the lower socioeconomic class according to the B.G. Prasad scale [10]. Overall, 219 (54.75%) participants had low physical activity, 66 (16.50%) had moderate physical activity, and 115 (28.75%) had high physical activity (Figure 1).

A statistically significant association was observed between gender and physical activity level (p<0.001). A higher proportion of females had low physical activity compared with males (143, 71.9% vs. 76, 37.8%). Females had significantly higher odds of low physical activity than males (OR=4.20; 95% CI: 2.75-6.40). Although the prevalence of low physical activity was higher among overweight (63, 56.3%) and obese (41, 60.3%) participants than those who were underweight (21, 52.5%) or had a normal BMI (94, 52.2%), this association was not statistically significant (p=0.29). No statistically significant associations were observed between physical activity level and age, education, occupation, or socioeconomic status (p>0.05 for all variables), as shown in Table 2. 

**Table 2:** Association of physical activity level with sociodemographic and anthropometric factors

Factor	Category	Low physical activity, n (%)	Moderate/high physical activity, n (%)	OR	95% CI	χ² (df)	p-value	Cramér's V
Gender	Male	76 (37.8)	125 (62.2)	1	—	46.79 (1)	<0.001	0.34
	Female	143 (71.9)	56 (28.1)	4.2	2.75-6.40			
Age group (years)	18-29	38 (52.1)	35 (47.9)	1	—	0.29 (3)	0.961	0.03
	30-44	74 (55.2)	61 (44.8)	1.12	0.63-1.98			
	45-59	61 (56.0)	48 (44.0)	1.17	0.65-2.11			
	≥60	46 (55.4)	37 (44.6)	1.15	0.62-2.13			
Education	Higher secondary and above	62 (54.4)	52 (45.6)	1	—	0.42 (2)	0.809	0.03
	Primary/illiterate	68 (57.1)	51 (42.9)	1.12	0.67-1.89			
	Secondary	89 (53.6)	78 (46.4)	0.96	0.58-1.59			
Occupation	Sedentary	54 (49.5)	55 (50.5)	1	—	3.52 (2)	0.172	0.09
	Homemaker	92 (60.5)	60 (39.5)	1.56	0.94-2.60			
	Manual worker	73 (52.5)	66 (47.5)	1.13	0.68-1.88			
Socioeconomic status	Upper	43 (53.8)	37 (46.2)	1	—	0.15 (2)	0.929	0.02
	Lower	79 (56.0)	62 (44.0)	1.1	0.64-1.90			
	Middle	97 (54.2)	82 (45.8)	1.02	0.61-1.70			
Body mass index	Normal	94 (52.2)	86 (47.8)	1	—	1.49 (3)	0.684	0.06
	Underweight	21 (52.5)	19 (47.5)	1.01	0.51-2.01			
	Overweight	63 (56.3)	49 (43.7)	1.18	0.73-1.91			
	Obese	41 (60.3)	27 (39.7)	1.39	0.79-2.45			

A total of 400 adults were included in the study, of whom 219 (54.75%) had low physical activity. Physical activity level was significantly associated with gender (χ²=46.79, df=1, p<0.001), with a moderate effect size (Cramér's V=0.34); women were more likely than men to be physically inactive. No statistically significant association was observed between physical activity level and age group (χ²=0.29, df=3, p=0.961; V=0.03), education (χ²=0.42, df=2, p=0.809; V=0.03), occupation (χ²=3.52, df=2, p=0.172; V=0.09), socioeconomic status (χ²=0.15, df=2, p=0.929; V=0.02), or BMI (χ²=1.49, df=3, p=0.684; V=0.06). The distribution of physical activity levels across the sociodemographic and anthropometric variables, with the corresponding test statistics, is presented in Table 2.

## Discussion

The present community‐based cross‐sectional study aimed to assess the prevalence and determinants of physical inactivity among adults residing in a rural region of central India. We found that 219 (54.75%) participants had low levels of physical activity, denoting a high prevalence of physical inactivity even in rural dwellers. A community‐based cross‐sectional study carried out in the villages of Uttar Pradesh reported an incidence of physical inactivity as 47.7% [11]. This corresponds to the gradual epidemiological transition in rural India, wherein previously active lifestyles are gradually being superseded by sedentary modes [12]. The prevalence reported in the present study is comparable with the recent estimates in other national and global studies. The pooled prevalence of insufficient physical activity among Indian adults steadily increased from 22.3% in 2000 to 49.4% in 2022, which was reported in a national study pooled from India in the Lancet Global Health [8]. In a study by Sharma et al., carried out in Chandigarh, the prevalence of inadequate physical activity was 52.1% [13]. A cross‐sectional study among medical students in Kashmir revealed an extremely low incidence of physical activity, at only 5.8% [14]. The trend appeared to have changed, and it may be concluded that the rural population is also not spared of the increasing burden of physical inactivity and its risk factors [15].

The present study observed a significant association between gender and physical activity, in which female subjects were significantly more likely to have low physical activity. Similar findings have been documented in a national population-based survey among 12,000 adults, where females had a higher prevalence of being physically inactive (52.4%) [16]. Several reports also mention low levels of leisure-time physical activity among women in several states of India, according to the ICMR-INDIAB study [7]. Similar findings have been reported in WHO reports and other community-based studies [9,12,17]. This difference could have been caused by sociocultural mores, unequal sharing of responsibilities among genders, paucity of time, fear of crime, social barriers, and limited access to open and leisure spaces, particularly in rural settings [18]. No significant association between BMI and physical activity was found in our study, although a higher proportion of overweight and obese individuals were physically inactive. Similar findings have been noted regarding low levels of physical activity [19]. The association between obesity and lack of physical activity is complex and may be bidirectional. 

Physical inactivity leads to a positive energy balance and weight gain, while increased body weight leads to lower levels of physical activity. According to us, the non-significance between BMI and physical activity may be due to the cross-sectional design of the study, where physical activity measures were self-reported, and to the influence of dietary and metabolic factors [20]. There was no significant association between levels of physical activity and age, education, occupation, or socioeconomic status [21]. While some studies reported an inverse or moderate association between them, other findings do not confirm the findings [11,13,16,22]. These findings suggest that the determinants of physical activity are multifactorial and are moderated by environmental, cultural, and infrastructural factors. The high prevalence of low physical activity represents a significant public health problem because physical inactivity is a major risk factor for cardiovascular diseases, diabetes mellitus, and some cancers. The public health challenge ultimately lies in promoting physical activity and should be the core of strategy for effective prevention and control of NCDs.

Strengths and limitations

Strengths of the current study were its community-based design and adequate sample size, which enhances the generalizability of the findings to other similar rural populations. The recent use of a validated method to assess physical activity also meant that the measurement of this component was standardized. However, the study did have some limitations. The cross-sectional nature of the study makes it difficult to determine whether physical activity can reliably predict the determinants uncovered. With physical activity assessed using a self-report measure, it is also possible that there was recall and social desirability bias influencing associations. Other determinants of physical activity that could act as confounders, such as dietary patterns, environmental influences, and metabolic variables, were not assessed in this study and could cloud the clarity of the associations presented.

## Conclusions

The study shows an overall high prevalence of inadequate physical activity among adults in a rural community of central India, even higher among females. Although BMI was not associated with physical activity, a disquieting observation was made with regard to low activity among the overweight and obese adults. The need for population-based interventions such as awareness programs in the community, promotion of an active lifestyle, designing of walkable streets and spaces, provision of facilities for physical activity in a safe ambiance, and inculcation of physical activity or exercise counseling into the primary healthcare services, especially for women in the community, cannot be emphasized enough in the context of the growing burden of NCDs.
